# Gastrointestinal Symptoms and Systemic Comorbidities in Patients With POTS: A Systematic Review and Meta‐Analysis

**DOI:** 10.1111/nmo.70305

**Published:** 2026-04-08

**Authors:** Dmitrii Kulin, Ayesha Shah, Thomas Fairlie, Kyle Staller, Samuel Nurko, Laurie Keefer, Qasim Aziz, Douglas A. Drossman, Michael P. Jones, Gerald Holtmann

**Affiliations:** ^1^ Faculty of Medicine, The University of Queensland Brisbane Queensland Australia; ^2^ Translational Research Institute Brisbane Queensland Australia; ^3^ Department of Gastroenterology & Hepatology Princess Alexandra Hospital Brisbane Queensland Australia; ^4^ Centre for Neurointestinal Health, Division of Gastroenterology, Massachusetts General Hospital Boston Massachusetts USA; ^5^ Harvard Medical School Boston Massachusetts USA; ^6^ Centre for Motility and Functional Gastrointestinal Disorders Boston Children's Hospital Boston Massachusetts USA; ^7^ Department of Pediatrics Harvard Medical School Boston Massachusetts USA; ^8^ Division of Gastroenterology Icahn School of Medicine at Mount Sinai New York City New York USA; ^9^ Centre for Neuroscience, Surgery and Trauma, Blizard Institute, Wingate Institute of Neurogastroenterology, Barts and The London School of Medicine and Dentistry Queen Mary University of London London UK; ^10^ Division of Gastroenterology University of N.C. and Drossman Gastroenterology Chapel Hill North Carolina USA; ^11^ School of Psychological Sciences Macquarie University North Ryde Australia

## Abstract

**Background:**

Patients with postural orthostatic tachycardia syndrome (POTS) frequently report higher rates of chronic gastrointestinal symptoms, disorders of gut‐brain interaction (DGBI), and extra‐intestinal co‐morbidities. We conducted a systematic review and meta‐analysis to assess the prevalence of gastrointestinal symptoms and comorbid conditions in POTS patients.

**Methods:**

Electronic databases were searched from inception until May 2025 for studies reporting gastrointestinal symptoms in POTS patients. A random‐effects model was used to pool the proportion of POTS patients reporting gastrointestinal symptoms, and sub‐group analyses were conducted.

**Results:**

The final dataset includes 19 studies, with 8268 POTS patients, revealing that 57.9% (95% CI 38.4–75.2) had at least one gastrointestinal symptom. The most common gastrointestinal symptom was nausea (70.1%, 95% CI 51.5–83.7) followed by bloating (64.9%, 95% CI 48.5–78.4), abdominal pain (60.4%, 95% CI 39.2–78.3) and postprandial fullness (60.4%, 95% CI 45.6–73.6). Irritable bowel syndrome was the most prevalent DGBI, affecting 26.8% (95% CI 15.3–42.4) of POTS patients. The most common extraintestinal comorbidity was anxiety, reported in 42.9% (95% CI 22.7–65.8), followed by chronic fatigue (40.9%, 95% CI 21.1–64.2), migraine (35.6%, 95% CI 27.0–45.2), depression (34.4%, 95% CI 19.0–54.0), and fibromyalgia (21.6%, 95% CI 12.8–34.2). Approximately one third reported mast cell activation syndrome (36.3%, 95% CI 17.8–60.0) and joint hypermobility syndrome (31%, 95% CI 24.4–38.5). There was substantial heterogeneity seen in the primary and most subgroup analyses.

**Conclusions:**

Overall, 60% of POTS patients report concurrent gastrointestinal symptoms, with nausea being the most common. IBS affects 25% of patients with POTS. Notably, extra‐intestinal comorbidities—primarily anxiety, chronic fatigue, migraines, depression, and fibromyalgia—are more prevalent than gastrointestinal conditions in this population.

## Plain Language Summary

The findings of this systematic review and meta‐analysis suggest that 60% of patients with POTS experience gastrointestinal symptoms, with nausea being the most common, reported by 70% of patients. Other common gastrointestinal symptoms include bloating, abdominal pain, postprandial fullness, constipation, and diarrhea. The exact pathophysiology of gastrointestinal symptoms in POTS remains unknown; patients with POTS also face a significant burden of chronic extra‐intestinal comorbidities that often exceed the prevalence of gastrointestinal conditions. Over one‐third report anxiety as the most common comorbidity, followed by chronic fatigue, migraines, depression, and fibromyalgia. Notably, IBS is the most common gastrointestinal disorder, affecting 25% of patients with POTS. Finally, autoimmune conditions are also more prevalent in POTS compared to the general population. While this systematic review and meta‐analysis is consistent with a link between POTS and GI symptoms or DGBI, the precise mechanisms for these associations need to be defined before causality can be assumed.

## Graphical abstract

This meta‐analysis reveals that a significant proportion of patients with POTS experience gastrointestinal symptoms. The most prevalent symptoms include nausea, bloating, abdominal pain, and postprandial fullness. There is a notable overlap of POTS with IBS, joint hypermobility, mast cell activation syndrome, and chronic fatigue.

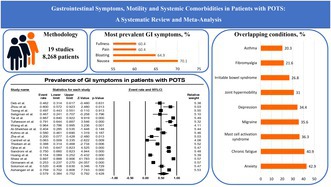

## 1 Introduction

Postural orthostatic tachycardia syndrome (POTS) is one of the most prevalent presentations of orthostatic intolerance, predominantly affecting young premenopausal women, with a reported prevalence rate of 0.2% [1] in 2015. The current prevalence rates of POTS are not known; however, increased awareness of the syndrome may have led to a rise in diagnosed cases over the past decade. It is defined as a clinical syndrome lasting at least 6 months characterized by an increase in heart rate of ≥ 30 beats/min (or ≥ 40 beats/min or more in children), often with a standing heart rate of > 120 beats/min, within 10 min of standing in adults or head‐up tilt (HUT), and in the absence of orthostatic hypotension [2]. POTS is typically associated with symptoms of orthostatic intolerance, including lightheadedness, dizziness, blurring or fading of vision, generalized weakness, fatigue, palpitations, mental clouding, anxiety, nausea, dyspnoea, or headache [2].

POTS is a syndrome, not a disease, often associated with various other symptoms and comorbidities that cannot physiologically be explained by orthostatic intolerance or tachycardia [1]. Patients with POTS often have a higher prevalence of various comorbidities, including chronic fatigue syndrome, syncope, migraines, fibromyalgia, joint hypermobility, Ehlers‐Danlos Syndrome, hypervigilance, anxiety, concussions, and autoimmune disorders [3]. It is speculated that these conditions may cluster together, suggesting the possibility of underlying shared pathophysiological mechanisms that link them with POTS. Moreover, an increasing number of recent studies have suggested an association between POTS and disabling chronic gastrointestinal symptoms with no underlying organic pathology that likely meet the criteria for disorders of gut‐brain interaction (DGBI), previously known as functional gastrointestinal disorders (FGID) [4]. Dysphagia, reflux, nausea, abdominal pain, bloating, and altered bowel habits are reported by up to 80% of subjects with POTS [5, 6]. Many symptoms occur independently of POTS, and some persist even after postural intolerance resolves, leaving the connection between these symptoms and POTS unclear. Moreover, symptoms can be severe, potentially leading to a significant reduction in quality of life, especially given management challenges [7].

The etiology of gastrointestinal symptoms in POTS is not entirely clear, multifactorial, potentially being either persistent or related to orthostatic intolerance. Whether it is related to primary underlying dysautonomia [5], the effects of dysautonomia on gastrointestinal motility [8], secondary to decompensation caused by POTS [9], autoimmunity [10], or due to comorbid disease [11] remains unclear. Gastrointestinal symptoms in patients with POTS, which are persistent, may also be caused by factors such as visceral hypersensitivity, central sensitization, psychological stress, and behavioral amplification [11].

Against this background, we performed a systematic review and meta‐analysis to assess the prevalence of chronic, gastrointestinal symptoms in patients with POTS, as well as identifying the most common gastrointestinal symptom within this patient population. Our secondary objective involved assessing the prevalence of commonly occurring gastrointestinal comorbidities and other concurrent comorbidities among patients diagnosed with POTS.

## 2 Materials and Methods

This systematic review and meta‐analysis meets the preferred reporting items for systematic reviews and meta‐analysis statement requirements (PRISMA) [12, 13]. The protocol for this Systematic Review was prospectively registered with PROSPERO (registration number CRD42025635134).

### 2.1 Search Strategy

**FIGURE 1:**
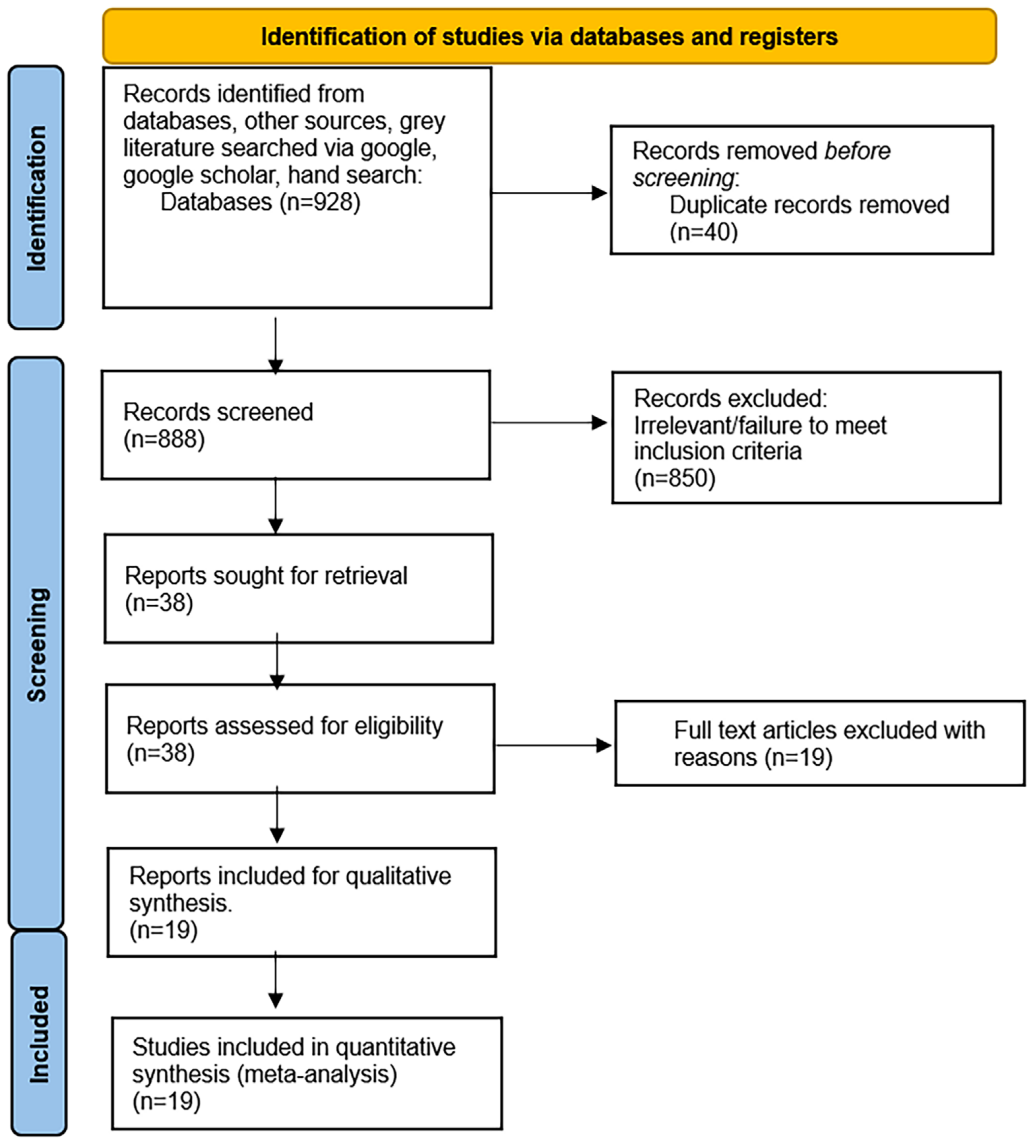
PRISMA flow diagram.

A comprehensive search of electronic databases, including MEDLINE (PubMed), EMBASE Cochrane, Web of Science, and Scopus was conducted from inception until 1 April 2025 for all studies reporting on both adult and pediatric patients with POTS and gastrointestinal symptoms. The detailed literature search strategy is outlined in the PRISMA flow diagram (Figure 1) and was conducted with the assistance of a librarian. The search strategy for MEDLINE has been outlined in the Supporting Information (Table S1). The initial search was not limited to specific languages to capture all appropriate studies. Gray literature was searched with Google and Google Scholar, and the “Snowball” method was also utilized to identify all relevant articles.

### 2.2 Selection of Studies

Three authors (DK, Ayesha Shah and TF) independently conducted an initial screening of abstracts and titles. Abstracts were eliminated if they did not meet the research topic. Full texts of the remaining articles were retrieved and reviewed. The studies that were excluded are provided in the Supporting Information (Table S2).

Eligible studies included cross‐sectional studies recruiting unselected subjects with POTS and reporting gastrointestinal symptoms. We included only those studies where the diagnosis of POTS was based on the clinical assessment, questionnaire data, or specific symptom‐based criteria, including HUT test, quantitative sudomotor axon reflex test (QSART), composite autonomic scoring scale (CASS), composite autonomic symptom scale (COMPASS), and tilt table test. Studies not reporting on original data, those reporting on mixed populations of functional disorders and subjects with organic diseases, studies with no separate data on subjects with POTS or those not reporting gastrointestinal manifestations in patients with POTS, as well as studies focusing on the prevalence of POTS in patients with gastrointestinal manifestations, were excluded. Conference abstracts that provided available data were also included in the study. Disagreements between reviewers were resolved by mutual consensus.

### 2.3 Data Extraction and Quality Assessment

Data extracted were entered into a Microsoft Excel spreadsheet (2016 Professional edition: Microsoft Corp, Redmond, Washington, USA). The following data were extracted: author, year of study, study design, country, mean age, gender, diagnostic criteria, inclusion/exclusion criteria of study, the numbers of patients with POTS with gastrointestinal symptoms, the number of patients with overlapping comorbid conditions, both gastrointestinal and extra‐intestinal.

The quality of the prevalence studies included was assessed by using the Joanna Briggs Institute (JBI) critical appraisal tools for use in JBI systematic reviews for prevalence studies [14], with the details provided in the Supporting Information (Table S4). The risk of bias was ranked as high when the study reached up to 49% of “yes” score, moderate when the study reached from 50% to 69% of “yes” score, and low when the study reached over 70% of “yes” score.

### 2.4 Data Synthesis and Statistical Analysis

In an initial step, case numbers of patients with POTS with gastrointestinal symptoms were extracted. As a second step, the pooled prevalence rates and 95% confidence intervals (CI) for the prevalence of gastrointestinal symptoms in patients with POTS were calculated. Subgroup analyses stratified according to specific gastrointestinal symptoms, and an overlap between POTS and gastrointestinal/extraintestinal comorbidities and the link between gastrointestinal function and POTS were performed. Finally, we did sensitivity analysis including only high‐quality studies (assessed utilizing the JBI critical appraisal tool), reporting the prevalence of gastrointestinal symptoms in patients with POTS.

Analysis for the prevalence of gastrointestinal symptoms in patients with POTS was carried out using the Comprehensive Meta‐Analysis (CMA) software version 4 (Biostat Inc., Englewood, NJ, USA). Logit transformation of proportions and the variance of the logit were used in the calculations to estimate pooled event rates and 95% confidence intervals (CIs) within groups and to compare event rates between groups. Pooled estimates of overlap prevalence were estimated using the DerSimonian and Laird random effects model to account for expected systematic between‐study variability appropriately.

Variability due to between‐study variation was evaluated using Cochrane's test and was quantified through the *I* ^2^ index. Further, we considered that either *χ* ^2^ test *p* < 0.10 or *I* ^2^ > 50% indicated substantial heterogeneity, *I* ^2^ > 75% indicated considerable heterogeneity, and *I* ^2^ > 30% to *I* ^2^ < 60% indicated moderate heterogeneity [15]. Standard approaches (Egger's test and visual inspection of funnel plots; SE versus log[odds], sample size vs. log[odds]) were applied to identify potential publication biases [16].

## 3 Results

### 3.1 Study Selection

The initial search revealed 928 publications. Of these, 38 published articles appeared to be relevant to the study question and were retrieved for further evaluation. Out of those, 19 were eligible for inclusion in this systematic review and meta‐analysis (Figure 1 and Table S2). The characteristics of all the studies in the current meta‐analysis, including the methodology pertaining to diagnosis of POTS and DGBI, patient characteristics are outlined in Table 1, and in the Supporting Information (Tables S3–S6).

**TABLE 1:** Characteristics of the studies included in the meta‐analysis.

	Study design	Total eligible participants, *n*	Mean age, years (SD), [age range]	POTS patients with GI complaints, *n*	POTS patients with nausea, *n*	POTS patients with abdominal pain, *n*	POTS patients with vomiting, *n*	POTS patients with constipation, *n*	POTS patients with bloating, *n*	POTS patients with postprandial fullness, *n*	POTS patients with diarrhea, *n*
Deb et al. [9]	Retrospective, cross‐sectional	39	35 (12) [NA]	18	NA	NA	NA	NA	NA	NA	NA
Tseng et al. [17]	Retrospective, cross‐sectional	332	29.3 (9.5) [NA]	165	165	145	NA	112	NA	NA	102
Seligman et al. [18]	Prospective, cross‐sectional	15	27 (4) [NA]	7	NA	NA	NA	NA	NA	NA	NA
Zhou et al. [7]	Retrospective, case–control	20	34 (2.6) [NA]	16	16	14	NA	15	15	NA	6
Tai et al. [5]	Cross‐sectional	231	37 (13) [NA]	205	127	187	42	NA	194	152	NA
Tufvesson et al. [19]	Case–control	43	30.6 (NA) [26–41]	34	34	NA	6	28	28	30	23
Wang et al. [8]	Cross‐sectional	28	30 (11) [NA]	27	24	20	NA	20	17	NA	NA
Al‐Shekhlee et al. [20]	Retrospective, cross‐sectional	57	27 (13) [NA]	23	NA	NA	NA	NA	NA	NA	NA
Kohno et al. [21]	Prospective, cross‐sectional	69	28 (10.3)	40	NA	NA	NA	NA	NA	NA	NA
Zha et al. [22]	Prospective, cross‐sectional	20	33 (12.7) [16–62]	4	NA	NA	NA	NA	NA	NA	NA
Cantrell et al. [23]	Prospective, cross‐sectional	16	36.1 (9.2) [18–52]	1	NA	NA	NA	NA	NA	NA	NA
Ginnaram et al. [24]	Retrospective, cross‐sectional	2610	NA	660	NA	NA	NA	NA	NA	NA	NA
Solomon et al. [25]	Retrospective, cross‐sectional	75	NA	39	NA	NA	NA	NA	NA	NA	NA
Ashangari et al. [26]	Retrospective, cross‐sectional	249	32.2 (NA)	189	189	80	NA	125	128	NA	NA
Thieben et al. [27]	Retrospective, cross‐sectional	152	30.2 (10.3) [NA]	59	59	23	13	23	36	NA	27
Ojha et al. [28]	Prospective, cross‐sectional	94	24.9 (8) [NA]	70	12	34	NA	NA	NA	NA	NA
Sandroni et al. [29]	Prospective, cross‐sectional	108	28.9 (9.5) [NA]	81	73	67	27	54	78	50	81
Huang et al. [30]	Retrospective, cross‐sectional	78	32 (NA) [18–54]	12	NA	NA	NA	NA	NA	NA	NA
Shaw et al. [31]	Cross‐sectional	4032	NA	3618	3618	3357	NA	2845	3184	NA	2783

Abbreviations: GI, gastrointestinal; NA, not available; POTS, postural orthostatic tachycardia syndrome; SD, standard deviation.

### 3.2 Prevalence of Gastrointestinal Symptoms in Patients With POTS

**FIGURE 2:**
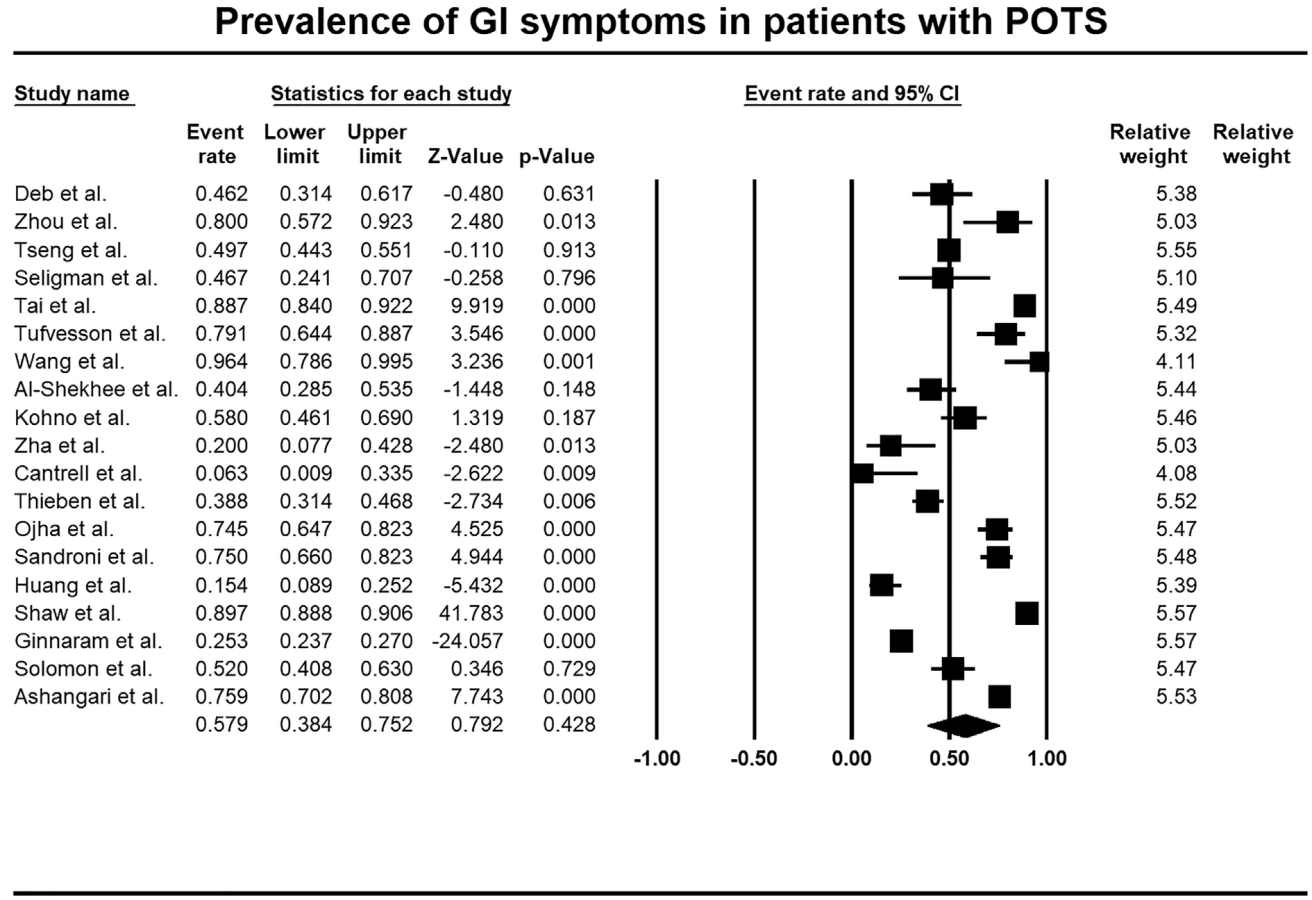
Forest plot of studies showing prevalence rates of gastrointestinal symptoms in patients with postural orthostatic tachycardia syndrome (POTS) (PP 57.9%, 95% CI 38.4–75.2, *I* ^2^ = 99.3, *p* < 0.001).

Overall, 19 studies [5, 7, 8, 9, 17, 19, 22, 23, 24, 25, 26] with 8268 adult patients (mean age ranging from 24.9 ± 8 to 37 ± 13 year, Table 1) with POTS included in this systematic review and meta‐analysis reported gastrointestinal symptoms. One [25] pediatric study met the inclusion criteria (Table S2). The prevalence of gastrointestinal symptoms in POTS patients was 57.9% (95% CI, 38.4–75.2, Figure 2), with considerable heterogeneity in the analysis (*I* ^2^ = 99.3, *p* < 0.001). Visual inspection of the funnel plot did not show any asymmetry (Figure S1), in keeping with the results of the Egger's test (*p* = 0.9), indicating no evidence of publication bias.

### 3.3 Influence of Risk of Bias on the Prevalence of Gastrointestinal Complaints in Patients With POTS

#### 3.3.1 Studies With Low Risk of Bias

**FIGURE 3:**
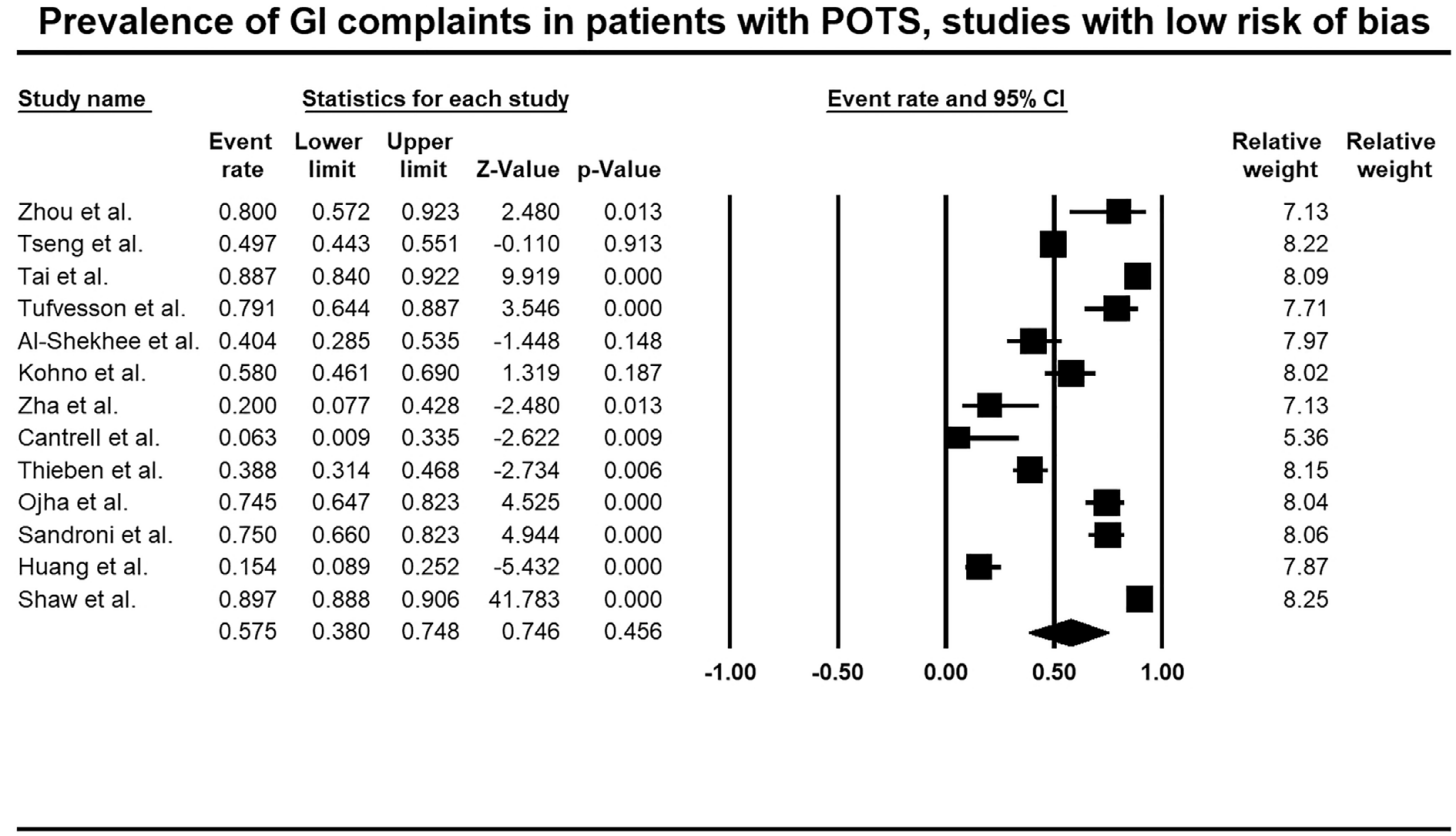
Forest plot of studies showing prevalence rates of gastrointestinal symptoms in patients with postural orthostatic tachycardia syndrome (POTS), including only high‐quality studies (PP 57.5%, 95% CI, 38.0–74.8, *I* ^2^ = 98.3, *p* < 0.001).

The majority (13/19, 68.4%) of the studies were of high quality, as assessed utilizing the JBI critical appraisal tool (Table S4). Including only thirteen high‐quality studies [5, 7, 17, 19, 22, 23], the prevalence of gastrointestinal complaints was estimated at 57.7% (95% CI, 38.0–74.8) of patients with POTS, with considerable heterogeneity seen in the studies included in the analysis (*I* ^2^ = 98.3, *p* < 0.001). Thus, conducting a sensitivity analysis did not reduce the heterogeneity in the studies included in the analyses (Figure 3, Table 2).

**TABLE 2:** Summary of findings of the outcomes reported in this systematic review and meta‐analysis.

	Number of studies, *n*	Number of eligible participants, *n*	Pooled prevalence, % (95% CI)	Assessment of heterogeneity between studies
Prevalence of GI complaints in patients with POTS	19	8268	57.9 (38.4–75.2)	*I* ^2^ = 99.3; *p* < 0.001
Prevalence of GI complaints in patients with POTS (low risk of bias)	13	5252	57.5 (38.0–74.8)	*I* ^2^ = 98.3; *p* < 0.001
Prevalence of GI complaints in patients with POTS diagnosed based on current criteria	14	1072	52.6 (41.0–63.9)	*I* ^2^ = 89.8; *p* < 0.001
Prevalence of GI complaints in patients with POTS in secondary care	5	309	42.5 (18.5–70.8)	*I* ^2^ = 92.6; *p* < 0.001
Prevalence of GI complaints in patients with POTS in tertiary care	11	1086	58.6 (47.7–68.6)	*I* ^2^ = 88.9; *p* < 0.001
Prevalence of nausea in patients with POTS	10	5215	70.1 (51.5–83.7)	*I* ^2^ = 98.5; *p* < 0.001
Prevalence of vomiting in patients with POTS	4	534	16.0 (10.2–24.2)	*I* ^2^ = 76.1; *p* = 0.006
Prevalence of abdominal pain in patients with POTS	9	5195	60.4 (39.2–78.3)	*I* ^2^ = 98.8; *p* < 0.001
Prevalence of bloating in patients with POTS	8	4863	64.9 (48.5–78.4)	*I* ^2^ = 97.3; *p* < 0.001
Prevalence of postprandial fullness in patients with POTS	3	382	60.4 (45.6–73.6)	*I* ^2^ = 84.7; *p* = 0.001
Prevalence of constipation in patients with POTS	8	4964	52.6 (36.0–68.6)	*I* ^2^ = 97.8; *p* < 0.001
Prevalence of diarrhea in patients with POTS	6	4687	45.6 (25.2–67.6)	*I* ^2^ = 98.3; *p* < 0.001
Prevalence of IBS in patients with POTS	12	7596	26.8 (15.3–42.4)	*I* ^2^ = 98.4; *p* < 0.001
Prevalence of GORD in patients with POTS	3	2879	9.9 (1.9–38.8)	*I* ^2^ = 95.4; *p* < 0.001
Prevalence of reported JHS in patients with POTS	8	4418	31.0 (24.4–38.5)	*I* ^2^ = 73.7; *p* < 0.001
Prevalence of asthma in patients with POTS	3	3996	20.3 (19.1–21.5)	*I* ^2^ < 0.1; *p* = 0.838
Prevalence of anxiety in patients with POTS	4	388	42.9 (22.7–65.8)	*I* ^2^ = 78.0; *p* = 0.003
Prevalence of chronic fatigue in patients with POTS	5	4668	40.9 (21.1–64.2)	*I* ^2^ = 98.8; *p* < 0.001
Prevalence of depression in patients with POTS	4	388	34.4 (19.0–54.0)	*I* ^2^ = 70.9; *p* = 0.016
Prevalence of fibromyalgia in patients with POTS	5	4532	21.6 (12.8–34.2)	*I* ^2^ = 94.8; *p* < 0.001
Prevalence of Hashimoto's disease in patients with POTS	3	3969	8.3 (4.1–16.0)	*I* ^2^ = 50.0; *p* = 0.136
Prevalence of reported MCAS in patients with POTS	3	4022	36.3 (17.8–60.0)	*I* ^2^ = 93.2; *p* < 0.001
Prevalence of migraine in patients with POTS	10	4669	35.6 (27.0–45.2)	*I* ^2^ = 90.6; *p* < 0.001
Prevalence of Raynaud's syndrome in patients with POTS	3	3969	15.5 (14.4–16.7)	*I* ^2^ < 0.1; *p* = 0.447
Prevalence of Sjogrens's syndrome in patients with POTS	3	3969	4.7 (1.7–12.2)	*I* ^2^ = 56.7; *p* = 0.099

Abbreviations: CI, confidence interval; GI, gastrointestinal; GORD, gastro‐esophageal reflux disease; IBS, irritable bowel syndrome; JHS, joint hypermobility syndrome; MCAS, mast cell activation syndrome; POTS, postural orthostatic tachycardia syndrome.

### 3.4 Influence of the Diagnostic Approach on the Prevalence of Gastrointestinal Complaints in Patients With POTS

#### 3.4.1 Studies Meeting Current Diagnostic Criteria for POTS

The majority (14/19, 73.7%) of the studies diagnosed POTS based on the current diagnostic criteria. Including only those studies, the prevalence of gastrointestinal symptoms was estimated at 52.6% (95% CI, 41.0–63.9) of patients with POTS, with considerable heterogeneity seen in the analysis (*I* ^2^ = 89.8, *p* < 0.001) (Figure S10, Table 2).

### 3.5 Influence of the Mode of Care on the Prevalence of Gastrointestinal Complaints in Patients With POTS

We conducted a subgroup analysis based on the mode of care (secondary/tertiary), from which patients were recruited. There was no statistically significant difference between modes of care (*p* = 0.315), with the prevalence of gastrointestinal symptoms in patients with POTS in tertiary care (58.6%, 95% CI 47.7–68.6) being higher than in secondary care (42.5%, 95% CI 18.5–70.8) (Figure S11). There was considerable heterogeneity seen in the analyses (Table 2).

### 3.6 Most Prevalent Gastrointestinal Symptoms in Patients With POTS

Nausea was the most prevalent gastrointestinal symptom reported in patients with POTS, occurring in 70.1% (95% CI 51.5–83.7) of patients based on data from 10 studies with 5215 subjects (Figure S2). Visual inspection of the funnel plot showed asymmetry (Figure S3), yet no clear evidence of bias was indicated by the results of the Egger's test (*p* = 0.13).

Following nausea, the second most prevalent gastrointestinal symptom was bloating, experienced by 64.9% (95% CI 48.5–78.4, 8 studies, *n* = 4863, Figure S4) of patients with POTS, followed by abdominal pain at 60.4% (95% CI: 39.2–78.3.8 studies, *n* = 4863, Figure S5) and postprandial fullness at 60.4% (95% CI: 45.6–73.6, 3 studies, *n* = 382, Figure S6).

Constipation was reported by 52.6% of patients with POTS (95% CI: 36.0–68.6, 8 studies, *n* = 4964, Figure S7), higher than diarrhea at 45.6% (95% CI: 25.2–67.6, 6 studies, *n* = 4687, Figure S8). The least common symptom was vomiting, found in 16% of POTS patients (95% CI: 10.2–24.2,4 studies, *n* = 534, Figure S9). There was considerable heterogeneity seen across all the analyses (Table 2).

### 3.7 Prevalence of DGBI and Other Gastrointestinal Comorbidities in Patients With POTS

**FIGURE 4:**
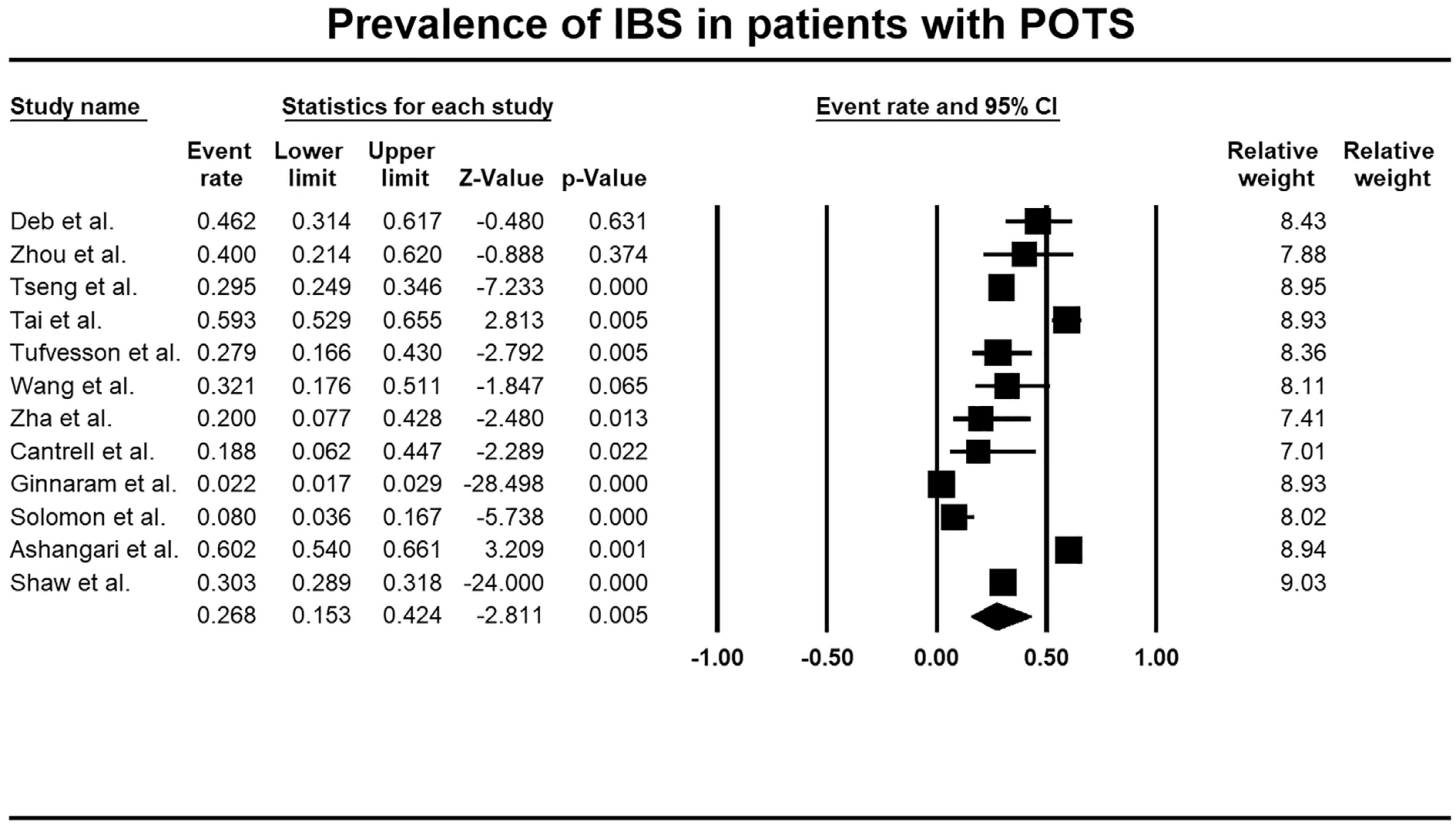
Forest plot of studies showing prevalence rates of irritable bowel syndrome (IBS) in patients with postural orthostatic tachycardia syndrome (POTS) (PP 26.8%, 95% CI, 15.3–42.4, *I* ^2^ = 98.4, *p* < 0.001).

Irritable bowel syndrome (IBS) was the most common gastrointestinal comorbidity among POTS patients, at 26.8% (95% CI: 15.3–42.4, 12 studies, *n* = 7596, Figure 4; Figure S12). Gastro‐esophageal reflux disease (GORD) followed, affecting 9.9% (95% CI: 1.9–38.8, 3 studies, *n* = 2879; Figure S13). Both analyses demonstrated considerable heterogeneity (Table 2). The mode of diagnosis of DGBI is outlined in Table S3.

### 3.8 Prevalence of Extraintestinal Comorbidities in Patients With POTS

Among patients with POTS, the most prevalent extra‐intestinal comorbidity was anxiety, affecting 42.9% (95% CI: 22.7–65.8, 4 studies, *n* = 388, Figure S14). This was closely followed by chronic fatigue, observed in 40.9% of patients (95% CI: 21.1–64.2, 5 studies, *n* = 4668, Figure S15), and migraine, which had a prevalence of 35.6% (95% CI: 27.0–45.2, 10 studies, *n* = 4669, Figures S17 and S18). Additionally, depression was reported in 34.4% of patients (95% CI: 19.0–54.0, 4 studies, *n* = 388, Figure S19), while fibromyalgia was seen in 21.6% (95% CI: 12.8–34.2, 5 studies, *n* = 4532, Figure S21).

Mast cell activation syndrome (MCAS) was reported in 36.3% of patients with POTS (95% CI: 17.8–60.0, 3 studies, *n* = 4022, Figure S16), followed by joint hypermobility syndrome (JHS) in 31% (95% CI: 24.4–38.5, 8 studies, *n* = 4418, Figure S20, Table S6). Only 2 studies [22, 31] reported an overlap of POTS with JHS and MCAS; however, data could not be extracted to determine how many patients had the overlap of all three conditions.

In patients with POTS, the prevalence of atopic and autoimmune conditions was as follows: asthma was reported in 20.3% (95% CI: 19.1–21.5, 3 studies, *n* = 3996, Figure S22) and Raynaud's syndrome was observed in 15.5% (95% CI: 14.4–16.7, 3 studies, *n* = 3969, Figure S23). Additionally, Hashimoto's disease was identified in 8.3% of patients with POTS (95% CI: 4.1–16.0, 3 studies, *n* = 3969, Figure S24), while Sjogren's syndrome affected 4.7% (95% CI: 1.7–12.2, 3 studies, *n* = 3969, Figure S25).

The heterogeneity noted in the studies included in these analyses was substantial, as reported in Table 2.

## 4 Discussion

This is the first systematic review and meta‐analysis reporting on the prevalence of gastrointestinal symptoms, gastrointestinal and extra‐intestinal co‐morbidities in patients with POTS. Overall, 57.9% of 8268 patients with pre‐existing POTS, included in 19 studies, had concurrent gastrointestinal symptoms. Nausea is the most prevalent gastrointestinal symptom seen in 70% of patients with POTS, followed closely by bloating, abdominal pain, postprandial fullness, constipation, and diarrhea. Abnormal gastric emptying and delayed colonic transit were observed in over one‐third of POTS patients studied. Extra‐intestinal comorbidities, including anxiety, chronic fatigue, migraines, and depression, were reported in over one‐third of POTS patients, occurring more frequently than gastrointestinal conditions. IBS is the most common DGBI affecting 25% of POTS patients. Additionally, autoimmune conditions such as asthma [32], Hashimoto's disease [33], Raynaud's syndrome [34], and Sjögren's syndrome [35] are more prevalent in POTS patients compared to the general population.

We noted substantial heterogeneity across the studies included in the primary analyses. The probable drivers of this clinical heterogeneity stem from the majority of studies being retrospective cohort designs conducted at tertiary centres, which introduces referral bias and a lack of comparison with healthy control groups. Additionally, comorbidities were self‐reported without adherence to established diagnostic criteria, and the studies employed variable diagnostic criteria for POTS. To address the heterogeneity, we conducted a sensitivity analysis including only high‐quality studies. However, this did not reduce the heterogeneity.

In addition to orthostatic symptoms, gastrointestinal symptoms are highly prevalent among POTS patients [4]. These symptoms can be severe, significantly impacting quality of life and presenting management challenges [7], resulting in increased healthcare utilisation [7, 17, 24, 36]. While the exact etiology of gastrointestinal symptoms in POTS remains unclear and likely multifactorial [4], the role of abnormal splanchnic blood flow is debated due to conflicting evidence [5]. Autonomic dysfunction [20, 37] and abnormal gastric myoelectrical activity [18, 38] seen in POTS may disrupt gastrointestinal function and motility. In an interesting study [39], out of 1925 patients presenting for autonomic function testing, patients reporting nausea were found to have decreased cerebral blood flow, which is associated with POTS. Additionally, factors such as posture‐dependent gastrointestinal symptoms [40], overlap with DGBI, psychological distress, central sensitization, and behavioral amplification [41] are frequently observed in POTS patients and may contribute to gastrointestinal symptoms. A recent review [42] has highlighted the role of increase in sympathetic nervous system (SNS) and reduced parasympathetic nervous system (PNS) tone that could result in delayed gastric emptying, visceral hypersensitivity, immune activation, and gastrointestinal symptoms in this subset of patients with POTS.

Patients with POTS face a significant burden of chronic extra‐intestinal comorbidities that often exceed the prevalence of gastrointestinal conditions. Over one‐third report anxiety as the most common comorbidity, followed by chronic fatigue, migraines, depression, and approximately 20% report fibromyalgia. Next, we looked at the prevalence of gastrointestinal conditions in patients with POTS. IBS is the most prevalent DGBI [43], with a global prevalence of 3.8% (95% CI 3.1–4.5) by Rome IV criteria to 9.2% (95% CI 7.6–10.8) by Rome III criteria [43]. Approximately one‐quarter of POTS patients also have IBS, indicating a significantly higher prevalence in this population. GORD affected 9.9% (95% CI 1.9–38.8) of the patients, significantly higher than the general population prevalence rates of 3.8% (95% CI 3.3–4.3) [44]. However, in most studies, diagnoses were based on clinical assessments documented in medical records, necessitating cautious interpretation of the data.

Moreover, POTS is also associated with a higher prevalence of atopic conditions and autoimmune disorders compared to the general population: Asthma (20.3% (95% CI 19.1–21.5) in POTS vs. 3.3% (95% CI 2.9–3.8) in the general population [32]), Hashimoto's disease (8.3% (95% CI 4.1–16.0) vs. 7.5% (95% CI 5.7–9.6) [33]), Raynaud's syndrome (15.5% (95% CI 14.4–16.7) vs. 4.85% (95% CI 2.1–8.7) [34]), and Sjögren's syndrome (4.7% (95% CI 1.7–12.2) versus 0.06% (95% CI 0.04–0.08) [35]).

Recently, the association between hypermobile Ehlers‐Danlos syndrome (hEDS), mast cell activation syndrome (MCAS), and postural orthostatic tachycardia syndrome (POTS) has gained attention, with more patients presenting this triad. A recent systematic review applying a vigorous literature search revealed limited original research on the triad, concluded that there was no current scientific evidence of any association between MCAS, POTS, or hEDS [45]. In our meta‐analysis, we found that approximately one third of POTS patients also report comorbid MCAS or hEDS. While these conditions may share overlapping symptoms, they are rare, and some would even question the true extent of their prevalence, due to different approaches to diagnostics and unclear pathophysiological mechanisms [45]; the overlap between all three independent variables could not be evaluated due to the limited data available, with the potential for further research focusing on evaluating the true prevalence of the triad.

Limited studies examined gastrointestinal motility in patients with POTS. Some have reported rapid gastric emptying [11, 46, 47] while others have reported delayed gastric emptying [7, 48] in patients with POTS, which could neither be confirmed nor refuted due to the limited number of studies. At this stage, further investigation is needed to substantiate the relationship between POTS and gastrointestinal motility.

A prior review [4], which included six studies with 352 patients with POTS, reported with 69% a similar pooled prevalence of gastrointestinal symptoms. Common symptoms included early satiety, nausea, bloating, and abdominal pain, which are associated with gastrointestinal motility abnormalities. The strength of the current study lies in our systematic assessment of the literature, which included over three times the number of studies in the previous review [4], and the ability to perform a meta‐analysis. Additionally, we thoroughly investigated potential explanations for the observed high heterogeneity. Furthermore, we conducted subgroup analyses to assess the prevalence of gastrointestinal and extra‐intestinal comorbidities in patients with POTS.

Some limitations of this meta‐analysis need to be acknowledged. The substantial to considerable between‐study heterogeneity observed in the primary analysis and the majority of the subgroup analyses could at least partially be explained by the inherent limitations and specifics of the studies included in this meta‐analysis, likely due to the lack of a control group, retrospective study design, and absence of uniform diagnostic criteria for POTS. The prevalence of comorbid conditions in patients with POTS reported in this systematic review and meta‐analysis may be skewed due to the majority of cases being self‐reported, including MCAS (3/4 studies relying on self‐reported data) and JHS (5/8 studies relying on self‐reported data). Consequently, some of the potentially existing comorbidities could have been either under‐ or overreported. The majority (11/19) of the studies were conducted in a tertiary care setting, introducing an element of selection bias limiting the generalisability of data. Quite importantly, there is no certainty whether the GI symptoms reported are pathophysiologically linked with POTS, given the high prevalence of comorbid conditions which may also present with gastrointestinal symptoms. The potential for the symptoms being linked to other comorbid conditions rather than POTS remains a possibility, and should be considered a potential confounding factor. Furthermore, it is worth noting the small sample size of studies, lack of a control group and small number of studies included in some subgroup analyses. As a result, the overall reliability of the available evidence is low, and the results need to be interpreted with caution.

In summary, the findings of this systematic review and meta‐analysis suggest that 60% of patients with POTS experience concurrent gastrointestinal symptoms, with nausea being the most common symptom reported by 70% of patients. Other common concurrent gastrointestinal symptoms include bloating, abdominal pain, postprandial fullness, constipation, and diarrhea. Although multifactorial, the pathophysiology of gastrointestinal symptoms in POTS remains unknown; patients with POTS also face a significant burden of chronic extra‐intestinal comorbidities that often exceed the prevalence of gastrointestinal conditions. Over one‐third report anxiety as the most common comorbidity, followed by chronic fatigue, migraines, depression, and fibromyalgia. Notably, IBS is the most common gastrointestinal disorder, affecting 25% of patients with POTS. Finally, autoimmune conditions are also more prevalent in POTS compared to the general population. While this systematic review and meta‐analysis is consistent with a link between POTS and GI symptoms or DGBI, the precise mechanisms for these associations need to be defined before causality can be assumed.

## Author Contributions

Dmitrii Kulin, Thomas Fairlie, Ayesha Shah, and Gerald Holtmann – study idea, concept and design, data extraction, data analysis and interpretation of data, and manuscript drafting. Michael P. Jones – data analysis, drafting of the manuscript, and review of the final manuscript. Laurie Keefer – drafting of the manuscript and review of the final manuscript. Douglas A. Drossman – drafting of the manuscript and review of the final manuscript. Qasim Aziz – drafting of the manuscript and review of the final manuscript. Samuel Nurko – drafting of the manuscript and review of the final manuscript. Kyle Staller – drafting of the manuscript and review of the final manuscript.

## Funding

National Health and Medical Research Council (APP2004495), Centre for Research Excellence (APP170993).

## Disclosure

Guarantor or article: Prof Gerald Holtmann.

## Conflicts of Interest

Gerald Holtmann reports to be on the advisory boards of Australian Biotherapeutics, Glutagen, Bayer and received research support from Bayer. He serves on the Boards of the West Moreton Hospital and Health Service (WMHHS), Queensland, UQ Healthcare, Brisbane and is Vice President of Gastro‐Liga, Germany and is Chair of the WMHHS Board Quality and Safety Committee. He serves as Editor of the Gastro‐Liga Newsletter. He is on the Research Committee of the Royal Australasian College of Physicians. Gerald Holtmann acknowledges funding from the National Health and Medical Research Council (NHMRC) for the Centre for Research Excellence in Digestive Health. Gerald Holtmann holds an MRFF and an NHMRC Ideas grant. Ayesha Shah acknowledges funding from the National Health and Medical Research Council (NHMRC) for the Centre for Research Excellence in Transforming Gut Health. Ayesha Shah holds an MRFF, NHMRC Ideas grant, and an NHMRC Emerging Leadership Fellow (EL1). Kyle Staller reports serving as a consultant to Anji, Ardelyx, Gemelli Biotech, Laborie, Mahana, ReStalsis, Salix, and Takeda. He has received research support from Ardelyx and ReStalsis. Laurie Keefer acknowledges research funds from Ardelyx and the Leona M and Harold B Helmsley Charitable Trust, and serves as a consultant for AbbVie, Pfizer, Eli Lilly, and J&J, and is an equity owner/co‐founder/advisor for Trellus Health. Samuel Nurko acknowledges research funds from Ardelyx and serves as a consultant for AbbVie and Ardelyx. Qasim Aziz serves on the medical advisory board for Ehlers Danlos Society, USA and has received a peer‐reviewed research grant from this society and the Ehlers Danlos Support Group UK.

## Supporting information


**Figure S1:** Funnel plot of studies showing prevalence of GI symptoms in patients with POTS.
**Figure S2:** Forest plot of studies showing prevalence rates of nausea in patients with POTS (PP 70.1%, 95% CI, 51.5–83.7, *I* ^2^ = 98.5, *p* < 0.001).
**Figure S3:** Funnel plot of studies showing prevalence of nausea in patients with POTS.
**Figure S4:** Forest plot of studies showing prevalence rates of bloating in patients with POTS (PP 64.9%, 95% CI, 48.5–78.4, *I* ^2^ = 97.3, *p* < 0.001).
**Figure S5:** Forest plot of studies showing prevalence rates of abdominal pain in patients with POTS (PP 60.4%, 95% CI, 39.2–78.3, *I* ^2^ = 98.8, *p* < 0.001).
**Figure S6:** Forest plot of studies showing prevalence rates of postprandial fullness in patients with POTS (PP 60.4%, 95% CI, 45.6–73.6, *I* ^2^ = 84.7, *p* = 0.001).
**Figure S7:** Forest plot of studies showing prevalence rates of constipation in patients with POTS (PP 52.6%, 95% CI, 36.0–68.6, *I* ^2^ = 97.8, *p* < 0.001).
**Figure S8:** Forest plot of studies showing prevalence rates of diarrhea in patients with POTS (PP 45.6%, 95% CI, 25.2–67.6, *I* ^2^ = 98.3, *p* < 0.001).
**Figure S9:** Forest plot of studies showing prevalence rates of vomiting in patients with POTS (PP 16%, 95% CI, 10.2–24.2, *I* ^2^ = 76.1, *p* = 0.006).
**Figure S10:** Forest plot of studies showing prevalence rates of GI symptoms in patients with POTS diagnosed based on current diagnostic criteria (PP 52.6%, 95% CI, 41.0–63.9, *I* ^2^ = 89.8, *p* < 0.001).
**Figure S11:** Forest plot of studies showing prevalence rates of GI symptoms in patients with POTS, stratified by mode of care.
**Figure S12:** Funnel plot of studies showing prevalence of IBS in patients with POTS.**Figure S13:** Forest plot of studies showing prevalence rates of GERD in patients with POTS (PP 9.9%, 95% CI, 1.9–38.8, *I* ^2^ = 95.4, *p* < 0.001).
**Figure S14:** Forest plot of studies showing prevalence rates of anxiety in patients with POTS (PP 42.9%, 95% CI, 22.7–65.8, *I* ^2^ = 78.0, *p* = 0.003).
**Figure S15:** Forest plot of studies showing prevalence rates of chronic fatigue in patients with POTS (PP 40.9%, 95% CI, 21.1–64.2, *I* ^2^ = 98.8, *p* < 0.001).
**Figure S16:** Forest plot of studies showing prevalence rates of reported MCAS in patients with POTS (PP 36.3%, 95% CI, 17.8–60.0, *I* ^2^ = 93.2, *p* < 0.001).
**Figure S17:** Forest plot of studies showing prevalence rates of migraine in patients with POTS (PP 35.6%, 95% CI, 27.0–45.2, *I* ^2^ = 90.6, *p* < 0.001).
**Figure S18:** Funnel plot of studies showing prevalence of migraine in patients with POTS.
**Figure S19:** Forest plot of studies showing prevalence rates of depression in patients with POTS (PP 34.4%, 95% CI, 19.0–54.0, *I* ^2^ = 70.9, *p* = 0.016).
**Figure S20:** Forest plot of studies showing prevalence rates of reported JHS in patients with POTS (PP 31.0%, 95% CI, 24.4–38.5, *I* ^2^ = 73.7, *p* < 0.001).
**Figure S21:** Forest plot of studies showing prevalence rates of fibromyalgia in patients with POTS (PP 21.6%, 95% CI, 12.8–34.2, *I* ^2^ = 94.8, *p* < 0.001).
**Figure S22:** Forest plot of studies showing prevalence rates of asthma in patients with POTS (PP 20.3%, 95% CI, 19.1–21.5, *I* ^2^ < 0.1, *p* = 0.838).
**Figure S23:** Forest plot of studies showing prevalence rates of Raynaud's syndrome in patients with POTS (PP 15.5%, 95% CI, 14.4–16.7, *I* ^2^ < 0.1, *p* = 0.447).
**Figure S24:** Forest plot of studies showing prevalence rates of Hashimoto's disease in patients with POTS (PP 8.3%, 95% CI, 4.1–16.0, *I* ^2^ = 49.9, *p* = 0.136).
**Figure S25:** Forest plot of studies showing prevalence rates of Sjogren's syndrome in patients with POTS (PP 4.7%, 95% CI, 1.7–12.2, *I* ^2^ = 56.7, *p* = 0.099).
**Table S1:** PubMed search strategy from University of Queensland librarian.
**Table S2:** Studies excluded from the systematic review and meta‐analysis with reasoning.
**Table S3:** Subject characteristics, inclusion and exclusion criteria for the studies included in the systematic review and meta‐analysis.
**Table S4:** Joanna Briggs Institute (JBI) critical appraisal for assessment of quality of studies included in this systematic review and meta‐analysis.
**Table S5:** Overlapping conditions in POTS.
**Table S6:** Joint hypermobility diagnostic approach.

## Data Availability

The data that support the findings of this study are available from the corresponding author upon reasonable request.
