# Supplementary material for: Gastrointestinal Symptoms and Systemic Comorbidities in Patients With POTS: A Systematic Review and Meta‐Analysis
Source: Neurogastroenterol Motil. 2026 Apr 8;38(4):e70305. doi: 10.1111/nmo.70305 (PMC13062037; doi:10.1111/nmo.70305)
Supplement: Supplementary file 1 — Figure S1: Funnel plot of studies showing prevalence of GI symptoms in patients with POTS. Figure S2: Forest plot of studies showing prevalence rates of nausea in patients with POTS (PP 70.1%, 95% CI, 51.5–83.7, I 2 = 98.5, p < 0.001). Figure S3: Funnel plot of studies showing prevalence of nausea in patients with POTS. Figure S4: Forest plot of studies showing prevalence rates of bloating in patients with POTS (PP 64.9%, 95% CI, 48.5–78.4, I 2 = 97.3, p < 0.001). Figure S5: Forest plot of studies showing prevalence rates of abdominal pain in patients with POTS (PP 60.4%, 95% CI, 39.2–78.3, I 2 = 98.8, p < 0.001). Figure S6: Forest plot of studies showing prevalence rates of postprandial fullness in patients with POTS (PP 60.4%, 95% CI, 45.6–73.6, I 2 = 84.7, p = 0.001). Figure S7: Forest plot of studies showing prevalence rates of constipation in patients with POTS (PP 52.6%, 95% CI, 36.0–68.6, I 2 = 97.8, p < 0.001). Figure S8: Forest plot of studies showing prevalence rates of diarrhea in patients with POTS (PP 45.6%, 95% CI, 25.2–67.6, I 2 = 98.3, p < 0.001). Figure S9: Forest plot of studies showing prevalence rates of vomiting in patients with POTS (PP 16%, 95% CI, 10.2–24.2, I 2 = 76.1, p = 0.006). Figure S10: Forest plot of studies showing prevalence rates of GI symptoms in patients with POTS diagnosed based on current diagnostic criteria (PP 52.6%, 95% CI, 41.0–63.9, I 2 = 89.8, p < 0.001). Figure S11: Forest plot of studies showing prevalence rates of GI symptoms in patients with POTS, stratified by mode of care. Figure S12: Funnel plot of studies showing prevalence of IBS in patients with POTS.Figure S13: Forest plot of studies showing prevalence rates of GERD in patients with POTS (PP 9.9%, 95% CI, 1.9–38.8, I 2 = 95.4, p < 0.001). Figure S14: Forest plot of studies showing prevalence rates of anxiety in patients with POTS (PP 42.9%, 95% CI, 22.7–65.8, I 2 = 78.0, p = 0.003). Figure S15: Forest plot of studies showing prevalence rates of chronic fatigue [file NMO-38-e70305-s001.docx]

**Supplementary material prepared for:** *Neurogastroenterology and Motility*

**Abbreviations used:**GI – gastrointestinal
POTS – postural orthostatic tachycardia syndrome

GE – gastric emptying

CTT – colonic transit time

IBS – irritable bowel syndrome

GERD – gastroesophageal reflux disease

MCAS – mast cell activation syndrome

JHS – joint hypermobility syndrome

**Legend of Figures:**

**Figure S1:** Funnel plot of studies showing prevalence of GI symptoms in patients with POTS

**Figure S2:** Forest plot of studies showing prevalence rates of nausea in patients with POTS (PP 70.1%, 95% CI, 51.5 - 83.7, I^2^=98.5, p<0.001).

**Figure S3:** Funnel plot of studies showing prevalence of nausea in patients with POTS

**Figure S4:** Forest plot of studies showing prevalence rates of bloating in patients with POTS (PP 64.9%, 95% CI, 48.5 – 78.4, I^2^=97.3, p<0.001).

**Figure S5:** Forest plot of studies showing prevalence rates of abdominal pain in patients with POTS (PP 60.4%, 95% CI, 39.2 – 78.3, I^2^=98.8, p<0.001).

**Figure S6:** Forest plot of studies showing prevalence rates of postprandial fullness in patients with POTS (PP 60.4%, 95% CI, 45.6 – 73.6, I^2^=84.7, p=0.001).

**Figure S7:** Forest plot of studies showing prevalence rates of constipation in patients with POTS (PP 52.6%, 95% CI, 36.0 – 68.6, I^2^=97.8, p<0.001).

**Figure S8:** Forest plot of studies showing prevalence rates of diarrhea in patients with POTS (PP 45.6%, 95% CI, 25.2 – 67.6, I^2^=98.3, p<0.001).

**Figure S9:** Forest plot of studies showing prevalence rates of vomiting in patients with POTS (PP 16%, 95% CI, 10.2 – 24.2, I^2^=76.1, p=0.006).

**Figure S10:** Forest plot of studies showing prevalence rates of GI symptoms in patients with POTS diagnosed based on current diagnostic criteria (PP 52.6%, 95% CI, 41.0 – 63.9, I^2^=89.8, p<0.001).

**Figure S11:** Forest plot of studies showing prevalence rates of GI symptoms in patients with POTS, stratified by mode of care

**Figure S12:** Funnel plot of studies showing prevalence of IBS in patients with POTS

**Figure S13:** Forest plot of studies showing prevalence rates of GERD in patients with POTS (PP 9.9%, 95% CI, 1.9 – 38.8, I^2^=95.4, p<0.001).

**Figure S14:** Forest plot of studies showing prevalence rates of anxiety in patients with POTS (PP 42.9%, 95% CI, 22.7 – 65.8, I^2^=78.0, p=0.003).

**Figure S15:** Forest plot of studies showing prevalence rates of chronic fatigue in patients with POTS (PP 40.9%, 95% CI, 21.1 – 64.2, I^2^=98.8, p<0.001).

**Figure S16:** Forest plot of studies showing prevalence rates of reported MCAS in patients with POTS (PP 36.3%, 95% CI, 17.8 – 60.0, I^2^=93.2, p<0.001).

**Figure S17:** Forest plot of studies showing prevalence rates of migraine in patients with POTS (PP 35.6%, 95% CI, 27.0 – 45.2, I^2^=90.6, p<0.001).

**Figure S18:** Funnel plot of studies showing prevalence of migraine in patients with POTS

**Figure S19:** Forest plot of studies showing prevalence rates of depression in patients with POTS (PP 34.4%, 95% CI, 19.0 – 54.0, I^2^=70.9, p=0.016).

**Figure S20:** Forest plot of studies showing prevalence rates of reported JHS in patients with POTS (PP 31.0%, 95% CI, 24.4 – 38.5, I^2^=73.7, p<0.001).

**Figure S21:** Forest plot of studies showing prevalence rates of fibromyalgia in patients with POTS (PP 21.6%, 95% CI, 12.8 – 34.2, I^2^=94.8, p<0.001).

**Figure S22:** Forest plot of studies showing prevalence rates of asthma in patients with POTS (PP 20.3%, 95% CI, 19.1 – 21.5, I^2^<0.1, p=0.838).

**Figure S23:** Forest plot of studies showing prevalence rates of Raynaud’s syndrome in patients with POTS (PP 15.5%, 95% CI, 14.4 – 16.7, I^2^<0.1, p=0.447).

**Figure S24:** Forest plot of studies showing prevalence rates of Hashimoto’s disease in patients with POTS (PP 8.3%, 95% CI, 4.1 – 16.0, I^2^=49.9, p=0.136).

**Figure S25:** Forest plot of studies showing prevalence rates of Sjogren’s syndrome in patients with POTS (PP 4.7%, 95% CI, 1.7 – 12.2, I^2^=56.7, p=0.099).

**Legend of Tables:**

**Table S1:** PubMed search strategy from University of Queensland librarian

**Table S2:** Studies excluded from the systematic review and meta-analysis with reasoning.

**Table S3:** Subject characteristics, inclusion and exclusion criteria for the studies included in the systematic review and meta-analysis.

**Table S4:** Joanna Briggs Institute critical appraisal for assessment of quality of studies included in this systematic review and meta-analysis.

**Table S5:** Overlapping conditions in POTS

**Table S6:** Joint hypermobility diagnostic approach

**Supplementary Figures:**

**Figure S1:** Funnel plot of studies showing prevalence of GI symptoms in patients with POTS

**Figure S2:** Forest plot of studies showing prevalence rates of nausea in patients with POTS (PP 70.1%, 95% CI, 51.5 - 83.7, I^2^=98.5, p<0.001).

**Figure S3:** Funnel plot of studies showing prevalence of nausea in patients with POTS

**Figure S4:** Forest plot of studies showing prevalence rates of bloating in patients with POTS (PP 64.9%, 95% CI, 48.5 – 78.4, I^2^=97.3, p<0.001).

**Figure S5:** Forest plot of studies showing prevalence rates of abdominal pain in patients with POTS (PP 60.4%, 95% CI, 39.2 – 78.3, I^2^=98.8, p<0.001).

**Figure S6:** Forest plot of studies showing prevalence rates of postprandial fullness in patients with POTS (PP 60.4%, 95% CI, 45.6 – 73.6, I^2^=84.7, p=0.001).

**Figure S7:** Forest plot of studies showing prevalence rates of constipation in patients with POTS (PP 52.6%, 95% CI, 36.0 – 68.6, I^2^=97.8, p<0.001).

`

**Figure S8:** Forest plot of studies showing prevalence rates of diarrhea in patients with POTS (PP 45.6%, 95% CI, 25.2 – 67.6, I^2^=98.3, p<0.001).

**Figure S9:** Forest plot of studies showing prevalence rates of vomiting in patients with POTS (PP 16%, 95% CI, 10.2 – 24.2, I^2^=76.1, p=0.006).

**Figure S10:** Forest plot of studies showing prevalence rates of GI symptoms in patients with POTS diagnosed based on current diagnostic criteria (PP 52.6%, 95% CI, 41.0 – 63.9, I^2^=89.8, p<0.001).

**Figure S11:** Forest plot of studies showing prevalence rates of GI symptoms in patients with POTS, stratified by mode of care

**Figure S12:** Funnel plot of studies showing prevalence of IBS in patients with POTS

**Figure S13:** Forest plot of studies showing prevalence rates of GERD in patients with POTS (PP 9.9%, 95% CI, 1.9 – 38.8, I^2^=95.4, p<0.001).

**Figure S14:** Forest plot of studies showing prevalence rates of anxiety in patients with POTS (PP 42.9%, 95% CI, 22.7 – 65.8, I^2^=78.0, p=0.003).

**Figure S15:** Forest plot of studies showing prevalence rates of chronic fatigue in patients with POTS (PP 40.9%, 95% CI, 21.1 – 64.2, I^2^=98.8, p<0.001).

**Figure S16:** Forest plot of studies showing prevalence rates of reported MCAS in patients with POTS (PP 36.3%, 95% CI, 17.8 – 60.0, I^2^=93.2, p<0.001).

**Figure S17:** Forest plot of studies showing prevalence rates of migraine in patients with POTS (PP 35.6%, 95% CI, 27.0 – 45.2, I^2^=90.6, p<0.001).

**Figure S18:** Funnel plot of studies showing prevalence of migraine in patients with POTS

**Figure S19:** Forest plot of studies showing prevalence rates of depression in patients with POTS (PP 34.4%, 95% CI, 19.0 – 54.0, I^2^=70.9, p=0.016).

**Figure S20:** Forest plot of studies showing prevalence rates of reported JHS in patients with POTS (PP 31.0%, 95% CI, 24.4 – 38.5, I^2^=73.7, p<0.001).

**Figure S21:** Forest plot of studies showing prevalence rates of fibromyalgia in patients with POTS (PP 21.6%, 95% CI, 12.8 – 34.2, I^2^=94.8, p<0.001).

**Figure S22:** Forest plot of studies showing prevalence rates of asthma in patients with POTS (PP 20.3%, 95% CI, 19.1 – 21.5, I^2^<0.1, p=0.838).

**Figure S23:** Forest plot of studies showing prevalence rates of Raynaud’s syndrome in patients with POTS (PP 15.5%, 95% CI, 14.4 – 16.7, I^2^<0.1, p=0.447).

**Figure S24:** Forest plot of studies showing prevalence rates of Hashimoto’s disease in patients with POTS (PP 8.3%, 95% CI, 4.1 – 16.0, I^2^=49.9, p=0.136).

**Figure S25:** Forest plot of studies showing prevalence rates of Sjogren’s syndrome in patients with POTS (PP 4.7%, 95% CI, 1.7 – 12.2, I^2^=56.7, p=0.099).

**Supplementary Tables:**

**Table S1:** PubMed search strategy from University of Queensland librarian

| 1. gut-brain 2. brain-gut axis 3. Irritable bowel syndrome 4. "Functional dyspepsia" 5. "Functional constipation" 6. "Reflux hypersensitivity" 7. "Chronic bowel dysfunction" 8. "Functional bowel disorder" 9. "Functional abdominal pain disorder" 10. "Chronic nausea and vomiting syndrome" 11. "Motility disorder" 12. "Functional chest pain" 13. "Functional heartburn" 14. Globus 15. "Functional dysphagia" 16. "Postprandial distress syndrome" 17. Epigastric pain syndrome 18. Belching disorder 19. cyclic vomiting syndrome 20. "functional diarrhea" 21. "functional abdominal bloating" 22. "biliary pain" 23. "Functional gallbladder disorder" 24. "functional sphincter of Oddi disorder" 25. faecal incontinence 26. "functional defecation disorder" 27. "functional anorectal pain" 28. "gastrointestinal diseases"[MeSH Terms] 29. Postural orthostatic tachycardia syndrome 30. DaCosta’s syndrome 31. "soldier’s heart" 32. "mitral valve prolapse syndrome" 33. Neurocirculatory asthenia 34. Orthostatic intolerance 35. "orthostatic tachycardia" 36. "postural tachycardia syndrome" 37. (1 OR 2 OR 3 OR 4 OR 5 OR 6 OR 7 OR 8 OR 9 OR 10 OR 11 OR 12 OR 13 OR 14 OR 15 OR 16 OR 17 OR 18 OR 19 OR 20 OR 21 OR 22 OR 23 OR 24 OR 25 OR 26 OR 27) AND (29 OR 30 OR 31 OR 32 OR 33 OR 34 OR 35 OR 36) 38. 28 AND (29 OR 30 OR 31 OR 32 OR 33 OR 34 OR 35 OR 36)   Limit: 01/1960-01/2025  Filters applied: MEDLINE  PubMed: 668 |
| --- |

**Table S2:** Studies excluded from the systematic review and meta-analysis with reasoning.

| Study | Reason for exclusion |
| --- | --- |
| Fikree et al., 2017^1^ | Data irrelevant to the primary research question |
| Sahin et al., 2023^2^ | Data irrelevant to the primary research question |
| Safder et al., 2009^3^ | Data irrelevant to the primary research question |
| Chelimsky et al., 2009^4^ | Data irrelevant to the primary research question |
| Chelimsky et al., 2012^5^ | Data irrelevant to the primary research question |
| Chelimsky et al., 2019^6^ | Data irrelevant to the primary research question |
| Bharucha et al., 1993^7^ | Data irrelevant to the primary research question |
| Zhang et al., 2019^8^ | Data irrelevant to the primary research question |
| Zhang et al., 2022^9^ | Data irrelevant to the primary research question |
| Kovacic et al., 2014^10^ | Data irrelevant to the primary research question |
| Antiel et al., 2008^11^ | Presence of GI symptoms was an inclusion criterion |
| Manini et al., 2020^12^ | Presence of GI symptoms was an inclusion criterion |
| Park et al., 2013^13^ | Presence of GI symptoms was an inclusion criterion |
| Garcia et al., 2017^14^ | Presence of GI symptoms was an inclusion criterion |
| Loavenbruck et al., 2015^15^ | Presence of GI symptoms was an inclusion criterion |
| Moak et al., 2016^16^ | No data on POTS |
| Sullivan et al., 2005^17^ | No data on POTS |
| Lawal et al., 2007^18^ | No data on POTS |
| Parker et al., 2021^19^ | Case series with <10 cases |

GI – gastrointestinal; POTS – postural orthostatic tachycardia syndrome

**Table S3:** Subject characteristics, inclusion and exclusion criteria for the studies included in the systematic review and meta-analysis.

| **No** | **Author** | **Country** | **Females, n (%)** | **Clinical diagnostic criteria (POTS)** | **Clinical diagnostic criteria (GI pathology)** | **Inclusions** | **Exclusion criteria** |
| --- | --- | --- | --- | --- | --- | --- | --- |
| 1 | Deb et al.^20^ | USA | 34 (89.5) | Clinical diagnosis | Clinical diagnosis | Patients with POTS | NA |
| 2 | Tseng et al.^21^ | USA | 294 (88.6) | Provided list of diagnostic criteria | GE scintigraphy | Patients with POTS | Other OI subtypes |
| 3 | Seligman et al.^22^ | UK | 14 (93.3) | Provided list of diagnostic criteria | Clinical diagnosis | Patients with POTS | History of gastric, intestinal or colonic surgery |
| 4 | Zhou et al.^23^ | USA | 20 (100) | QSART, HUT test | Motility capsule testing | Patients who underwent autonomic testing and wireless motility capsule testing | Patients not eligible for wireless motility capsule test |
| 5 | Tai et al.^24^ | UK | 225 (97.4) | Clinical diagnosis | Rome IV | Hypermobility type Ehlers-Danlos syndrome patients | Organic GI disease |
| 6 | Tufvesson et al.^25^ | Sweden | 40 (93.0) | HUT test | Gastroparesis questionnaire, VAS-IBS, IBS-SSS | Patients with POTS | NA |
| 7 | Wang et al.^26^ | USA | 24 (92.3) | Clinical diagnosis | Clinical diagnosis | Patients with POTS | NA |
| 8 | Al-Shekhlee et al.^27^ | USA | 41 (71.9) | QSART, HUT test | Clinical diagnosis | Patients with POTS | Patients with transient early heart rate increase, patients with borderline findings, patients that could not tolerate testing |
| 9 | Kohno et al.^28^ | USA | 38 (95.0) | Provided list of diagnostic criteria | Clinical diagnosis | Patients with POTS | <4 months symptom duration, ongoing inflammatory disease, suspected volume depletion, declined consent |
| 10 | Zha et al.^29^ | USA | 20 (100) | TTT test, COMPASS | Clinical diagnosis | Patients with POTS maintaining a 4-week gluten free diet | Patients unable to maintain a gluten free diet |
| 11 | Cantrell et al.^30^ | USA | 13 (81.3) | TTT test, QSART, COMPASS, SPB, Valsalva | Clinical diagnosis | Patients infected with COVID-19 who were subsequently diagnosed with POTS | NA |
| 12 | Ginnaram et al.^31^ | USA | 1467 (56.2) | Clinical diagnosis | Clinical diagnosis | Patients with POTS | NA |
| 13 | Solomon et al.^32^ | USA | NA | Clinical diagnosis | Rome IV | Children with POTS and JHS/EDS | NA |
| 14 | Ashangari et al.^33^ | USA | 226 (90.8) | NA | NA | Patients with POTS | NA |
| 15 | Thieben et al.^34^ | USA | 132 (86.8) | Clinical diagnosis, with provided list of diagnostic criteria | NA | Patients with POTS | 1) Orthostatic hypotension defined as a decline of 30 mm Hg or more in systolic blood pressure or 20 mm Hg or more in mean blood pressure within 3 minutes of standing or head-up tilt 2) Pregnancy or lactation 3) Presence of another cause of autonomic failure 4) Presence of failure of other organ system or systemic illness affecting autonomic function or the patient’s ability to cooperate (dementia, pheochromocytoma, congestive heart failure, hypertension, renal or hepatic disease, severe anemia, alcoholism, malignant neoplasm, diabetes, hypothyroidism, sympathectomy, or cerebrovascular accident). |
| 16 | Ojha et al.^35^ | USA | 87 (82.1) | TTT test, ODYSA | NA | Patients with POTS | NA |
| 17 | Sandroni et al.^36^ | USA | 88 (81.5) | Clinical diagnosis, with provided list of diagnostic criteria | NA | Patients with POTS | 1) Orthostatic hypotension defined as a decline in systolic blood pressure of 30 mm Hg or more or in mean blood pressure of 20 mm Hg or more within 3 minutes of standing up or head-up tilt 2) Pregnancy or lactation 3) Presence of another cause of autonomic failure 4) Presence of failure of other organ systems or systemic illness that can affect autonomic function or the patient's ability to cooperate (dementia, pheochromocytoma, congestive heart failure, hypertension, renal or hepatic disease, severe anemia, alcoholism, malignant neoplasm, hypothyroidism, sympathectomy, or cerebrovascular accident) 5) Concomitant therapy with anticholinergic drugs, or other medications that could interfere with testing of autonomic function 6) Clinically significant coronary artery disease. |
| 18 | Huang et al.^37^ | USA | 11 (91.7) | Clinical diagnosis, with provided list of diagnostic criteria | NA | Patients with POTS | NA |
| 19 | Shaw et al.^38^ | USA | 4539 (93.9) | Self-reported clinical diagnosis | NA | Consenting patients who reported a clinical diagnosis of POTS | Non-consenting patients with POTS and patients without a diagnosis received from a physician |

GI – gastrointestinal; POTS – postural orthostatic tachycardia syndrome; NA – not available; OI – orthostatic intolerance; GE – gastric emptying; QSART – quantitative sudomotor axon reflex test; HUT – head up-tilt; VAS-IBS – visual analogue scale for irritable bowel syndrome; IBS-SSS – irritable bowel syndrome symptom severity scale; COMPASS – composite autonomic symptom scale; SPB – skin punch biopsy; TTT – table tilt test; ODYSA – Ohio dysautonomia survey
JHS – joint hypermobility syndrome; EDS – Ehlers-Danlos syndrome

**Table S4:** Joanna Briggs Institute (JBI) critical appraisal for assessment of quality of studies included in this systematic review and meta-analysis.

| No | Author | 1) sample  frame  appropriate  to address  target pop  (POTS  subjects) | 2)  participants  sampled in an  appropriate  way | 3) sample  size  adequate | 4) study  subjects  and  setting  details | 5) data  analysis  conducted  with  sufficient  coverage | 6) valid methods  for identifying  condition | 7) condition measured in  standard reliable way for  all participants | 8)  appropriate  statistical  analysis | 9)  response  rate  adequate  (>80%) | JBI  total | JBI  total  % |
| --- | --- | --- | --- | --- | --- | --- | --- | --- | --- | --- | --- | --- |
| 1 | Deb et al. | 1 | 1 | N | 1 | 1 | N | N | U | 1 | 5 | 55.6 |
| 2 | Tseng et al. | 1 | 1 | 1 | 1 | 1 | 1 | 1 | 1 | 1 | 9 | 100 |
| 3 | Seligman et al. | 1 | 1 | N | 1 | N | U | N | 1 | 1 | 5 | 55.6 |
| 4 | Zhou et al. | 1 | 1 | N | 1 | 1 | 1 | 1 | 1 | 1 | 8 | 88.9 |
| 5 | Tai et al. | 1 | 1 | 1 | 1 | 1 | U | 1 | 1 | N | 7 | 77.8 |
| 6 | Tufvesson et al. | 1 | 1 | N | 1 | 1 | 1 | 1 | 1 | N | 7 | 77.8 |
| 7 | Wang et al. | 1 | 1 | N | 1 | 1 | U | N | N | 1 | 5 | 55.6 |
| 8 | Al-Shekhlee et al. | 1 | 1 | N | 1 | 1 | U | 1 | 1 | 1 | 7 | 77.8 |
| 9 | Kohno et al. | 1 | 1 | N | 1 | 1 | U | N | 1 | 1 | 6 | 66.7 |
| 10 | Zha et al. | 1 | 1 | N | 1 | 1 | 1 | 1 | 1 | 1 | 8 | 88.9 |
| 11 | Cantrell et al. | 1 | 1 | N | 1 | 1 | 1 | N | U | 1 | 6 | 66.7 |
| 12 | Ginnaram et al. | 1 | 1 | 1 | N | U | U | 1 | U | U | 4 | 44.4 |
| 13 | Solomon et al. | 1 | 1 | N | N | U | 1 | 1 | U | U | 4 | 44.4 |
| 14 | Ashangari et al. | 1 | 1 | 1 | 1 | U | U | U | U | U | 4 | 44.4 |
| 15 | Thieben et al. | 1 | 1 | 1 | 1 | 1 | 1 | 1 | 1 | 1 | 9 | 100 |
| 16 | Ojha et al. | 1 | 1 | N | 1 | 1 | 1 | 1 | U | 1 | 7 | 77.8 |
| 17 | Sandroni et al. | 1 | 1 | 1 | 1 | 1 | 1 | 1 | U | 1 | 8 | 88.9 |
| 18 | Huang et al. | 1 | 1 | N | 1 | 1 | 1 | 1 | U | 1 | 7 | 77.8 |
| 19 | Shaw et al. | 1 | 1 | 1 | N | 1 | U | U | 1 | 1 | 6 | 66.7 |

POTS – postural orthostatic tachycardia syndrome; N – criterion not met; U – unavailable

**Table S5:** Overlapping conditions in POTS

|  | Total eligible participants, n | IBS, n | GERD, n | JHS, n | Asthma, n | Anxiety, n | Depression, n | Chronic fatigue, n | Fibromyalgia, n | MCAS, n | Hashimoto’s disease, n | Migraine, n | Raynaud’s syndrome, n | Sjogren’s syndrome, n |
| --- | --- | --- | --- | --- | --- | --- | --- | --- | --- | --- | --- | --- | --- | --- |
| Deb et al. | 39 | 18 | NA | 7 | NA | NA | NA | NA | NA | NA | NA | NA | NA | NA |
| Tseng et al. | 332 | 98 | NA | 100 | NA | 132 | 109 | 215 | 55 | NA | NA | 207 | NA | NA |
| Seligman et al. | 15 | NA | NA | 11 | NA | NA | NA | NA | NA | NA | NA | NA | NA | NA |
| Zhou et al. | 20 | 8 | NA | 10 | NA | 10 | 7 | 6 | 3 | NA | NA | 8 | NA | NA |
| Tai et al. | 231 | 137 | NA | NA | NA | NA | NA | 102 | 102 | NA | NA | NA | NA | NA |
| Tufvesson et al. | 43 | 12 | NA | 12 | 9 | NA | NA | NA | NA | NA | NA | 7 | NA | NA |
| Wang et al. | 28 | 9 | NA | NA | NA | NA | NA | NA | NA | NA | NA | NA | NA | NA |
| Al-Shekhlee et al. | 57 | NA | NA | NA | NA | NA | NA | NA | NA | NA | NA | NA | NA | NA |
| Kohno et al. | 69 | NA | NA | NA | NA | NA | NA | NA | NA | 29 | NA | 17 | NA | NA |
| Zha et al. | 20 | 4 | 2 | 8 | 3 | 1 | 1 | NA | NA | 11 | 3 | 2 | 1 | 1 |
| Cantrell et al. | 16 | 3 | NA | 2 | NA | 12 | 10 | NA | 1 | NA | 2 | 6 | 2 | 3 |
| Ginnaram et al. | 2610 | 58 | 660 | NA | NA | NA | NA | NA | NA | NA | NA | NA | NA | NA |
| Solomon et al. | 75 | 6 | NA | NA | NA | NA | NA | NA | NA | NA | NA | NA | NA | NA |
| Ashangari et al. | 249 | 150 | 8 | NA | NA | NA | NA | NA | NA | NA | NA | NA | NA | NA |
| Thieben et al. | 152 | NA | NA | NA | NA | NA | NA | 73 | NA | NA | NA | 42 | NA | NA |
| Ojha et al. | 44 | NA | NA | NA | NA | NA | NA | NA | NA | NA | NA | 20 | NA | NA |
| Sandroni et al. | 40 | NA | NA | NA | NA | NA | NA | NA | NA | NA | NA | 18 | NA | NA |
| Huang et al. | 78 | NA | NA | NA | NA | NA | NA | NA | NA | NA | NA | NA | NA | NA |
| Shaw et al. | 3933 | 1192 | NA | 994 | 798 | NA | NA | 809 | 786 | 353 | 228 | 1557 | 610 | 112 |

POTS – postural orthostatic tachycardia syndrome; IBS – irritable bowel syndrome; GERD – gastroesophageal reflux disease; JHS – joint hypermobility syndrome
MCAS – mast cell activation syndrome; NA – not available

**Table S6:** Joint hypermobility diagnostic approach

|  | Publication year | Condition reported | Diagnostic criteria |
| --- | --- | --- | --- |
| Deb et al. | 2015 | Ehlers-Danlos syndrome, Joint hypermobility syndrome | Unspecified |
| Zhou et al. | 2021 | Joint hypermobility syndrome | 2017 Ehlers Danlos Society Diagnostic Criteria |
| Tseng et al. | 2019 | Ehlers-Danlos syndrome type III | Beighton score |
| Seligman et al. | 2013 | Joint hypermobility syndrome, Ehlers-Danlos syndrome type III | Beighton score |
| Tufvesson et al. | 2024 | Hypermobile spectrum disorders/Ehlers-Danlos syndrome | Self-reported |
| Zha et al. | 2023 | Hypermobile Ehlers-Danlos syndrome | Unspecified |
| Cantrell et al. | 2024 | Ehlers-Danlos syndrome | Past medical history |
| Shaw et al. | 2019 | Ehlers-Danlos syndrome | Clinical diagnosis, unspecified |

**References:**

1. Fikree A, Aziz Q, Sifrim D. Mechanisms underlying reflux symptoms and dysphagia in patients with joint hypermobility syndrome, with and without postural tachycardia syndrome. Neurogastroenterol Motil 2017;29.

2. Sahin NU, Sahin N, Kilic M. Effect of comorbid benign joint hypermobility and juvenile fibromyalgia syndromes on pediatric functional gastrointestinal disorders. Postgrad Med 2023;135:386-393.

3. Safder S, Chelimsky TC, O'Riordan MA, et al. Autonomic testing in functional gastrointestinal disorders: implications of reproducible gastrointestinal complaints during tilt table testing. Gastroenterol Res Pract 2009;2009:868496.

4. Chelimsky G, Madan S, Alshekhlee A, et al. A comparison of dysautonomias comorbid with cyclic vomiting syndrome and with migraine. Gastroenterol Res Pract 2009;2009:701019.

5. Chelimsky G, Safder S, Chelimsky T. FGIDs in children are associated with many nonpsychiatric comorbidities: the tip of an iceberg? J Pediatr Gastroenterol Nutr 2012;54:690-1.

6. Chelimsky G, Rausch S, Bierer D, et al. Cardiovagal modulation in pediatric functional gastrointestinal disorders. Neurogastroenterol Motil 2019;31:e13564.

7. Bharucha AE, Camilleri M, Low PA, et al. Autonomic dysfunction in gastrointestinal motility disorders. Gut 1993;34:397-401.

8. Zhang LN, Moak JP, Desbiens J, et al. Utility of Diagnostic Studies for Upper Gastrointestinal Symptoms in Children with Orthostatic Intolerance. J Pediatr 2019;205:138-144.

9. Zhang R, Mayuga K, Shields R, et al. Skin Biopsy and Quantitative Sudomotor Axon Reflex Testing in Patients With Postural Orthostatic Tachycardia Syndrome. Cureus 2022;14:e31021.

10. Kovacic K, Chelimsky TC, Sood MR, et al. Joint hypermobility: a common association with complex functional gastrointestinal disorders. J Pediatr 2014;165:973-8.

11. Antiel RM, Risma JM, Grothe RM, et al. Orthostatic intolerance and gastrointestinal motility in adolescents with nausea and abdominal pain. J Pediatr Gastroenterol Nutr 2008;46:285-8.

12. Manini ML, Barazi A, Khemani D, et al. Gastrointestinal motility evaluation in children with orthostatic intolerance: Mayo Clinic experience. Neurogastroenterol Motil 2020;32:e13863.

13. Park KJ, Singer W, Sletten DM, et al. Gastric emptying in postural tachycardia syndrome: a preliminary report. Clin Auton Res 2013;23:163-7.

14. Garcia FE. Experience with an emerging clinical triad: Gastrointestinal dysmotility as assessed by comprehensive gastrointestinal scintigraphy in patients with joint hypermobility syndrome and dysautonomia. Digestive Disease Week 2017, DDW 2017. Volume 152:5. United States, Chicago, IL: Gastroenterology, 2017:S509.

15. Loavenbruck A, Iturrino J, Singer W, et al. Disturbances of gastrointestinal transit and autonomic functions in postural orthostatic tachycardia syndrome. Neurogastroenterol Motil 2015;27:92-8.

16. Moak JP, Fabian RR, Clarke LC, et al. Antroduodenal Manometry Is Abnormal in Children Presenting With Orthostatic Intolerance and Gastrointestinal Symptoms. J Pediatr Gastroenterol Nutr 2016;63:329-35.

17. Sullivan SD, Hanauer J, Rowe PC, et al. Gastrointestinal symptoms associated with orthostatic intolerance. J Pediatr Gastroenterol Nutr 2005;40:425-8.

18. Lawal A, Barboi A, Krasnow A, et al. Rapid gastric emptying is more common than gastroparesis in patients with autonomic dysfunction. Am J Gastroenterol 2007;102:618-23.

19. Parker WH, Moudgil R, Wilson RG, et al. COVID-19 and postural tachycardia syndrome: a case series. Eur Heart J Case Rep 2021;5:ytab325.

20. Deb A, Morgenshtern K, Culbertson CJ, et al. A survey-based analysis of symptoms in patients with postural orthostatic tachycardia syndrome. Proc (Bayl Univ Med Cent) 2015;28:157-9.

21. Tseng AS, Traub NA, Harris LA, et al. Factors Associated With Use of Nonoral Nutrition and Hydration Support in Adult Patients With Postural Tachycardia Syndrome. JPEN J Parenter Enteral Nutr 2019;43:734-741.

22. Seligman WH, Low DA, Asahina M, et al. Abnormal gastric myoelectrical activity in postural tachycardia syndrome. Clin Auton Res 2013;23:73-80.

23. Zhou W, Zikos TA, Clarke JO, et al. Regional Gastrointestinal Transit and Contractility Patterns Vary in Postural Orthostatic Tachycardia Syndrome (POTS). Dig Dis Sci 2021;66:4406-4413.

24. Tai FWD, Palsson OS, Lam CY, et al. Functional gastrointestinal disorders are increased in joint hypermobility-related disorders with concomitant postural orthostatic tachycardia syndrome. Neurogastroenterol Motil 2020;32:e13975.

25. Tufvesson H, Hamrefors V, Fedorowski A, et al. Gastrointestinal symptoms in patients with postural orthostatic tachycardia syndrome in relation to hemodynamic findings and immunological factors. Front Physiol 2024;15:1342351.

26. Wang LB, Culbertson CJ, Deb A, et al. Gastrointestinal dysfunction in postural tachycardia syndrome. J Neurol Sci 2015;359:193-6.

27. Al-Shekhlee A, Lindenberg JR, Hachwi RN, et al. The value of autonomic testing in postural tachycardia syndrome. Clin Auton Res 2005;15:219-22.

28. Kohno R, Cannom DS, Olshansky B, et al. Mast Cell Activation Disorder and Postural Orthostatic Tachycardia Syndrome: A Clinical Association. J Am Heart Assoc 2021;10:e021002.

29. Zha K, Brook J, McLaughlin A, et al. Gluten-free diet in postural orthostatic tachycardia syndrome (POTS). Chronic Illn 2023;19:409-417.

30. Cantrell C, Reid C, Walker CS, et al. Post-COVID postural orthostatic tachycardia syndrome (POTS): a new phenomenon. Front Neurol 2024;15:1297964.

31. Ginnaram S. GENDER-BASED IMPACT OF POTS ON GASTROINTESTINAL MOTILITY: A NATIONWIDE ANALYSIS. DDW 2024. Volume 166:5. United States, Washington, DC: Gastroenterology, 2024:S1071.

32. Solomon V. Presence of functional gastrointestinal disorders (FGIDs) in pediatric patients with ehlers-danlos syndrome or joint hypermobility syndrome and postural orthostatic tachycardia syndrome. North American Society for Pediatric Gastroenterology, Hepatology and Nutrition Annual Meeting, NASPGHAN 2021. Volume 73:1. Virtual: Journal of Pediatric Gastroenterology and Nutrition, 2021:S446-S437.

33. Ashangari C. Gastrointestinal disturbances in postural orthostatic tachycardia syndrome (POTS). International Academy of Cardiology 20th World Congress on Heart Disease Annual Scientific Sessions 2015. Volume 131 (304). Vancouver, BC, Canada: Cardiology (Switzerland), 2015:304.

34. Thieben MJ, Sandroni P, Sletten DM, et al. Postural orthostatic tachycardia syndrome: the Mayo clinic experience. Mayo Clin Proc 2007;82:308-13.

35. Ojha A, Chelimsky TC, Chelimsky G. Comorbidities in pediatric patients with postural orthostatic tachycardia syndrome. J Pediatr 2011;158:20-3.

36. Sandroni P, Opfer-Gehrking TL, McPhee BR, et al. Postural tachycardia syndrome: clinical features and follow-up study. Mayo Clin Proc 1999;74:1106-10.

37. Huang RJ, Chun CL, Friday K, et al. Manometric abnormalities in the postural orthostatic tachycardia syndrome: a case series. Dig Dis Sci 2013;58:3207-11.

38. Shaw BH, Stiles LE, Bourne K, et al. The face of postural tachycardia syndrome - insights from a large cross-sectional online community-based survey. J Intern Med 2019;286:438-448.
